# Comorbid Obsessive-Compulsive Symptoms in Schizophrenia: Insight into Pathomechanisms Facilitates Treatment

**DOI:** 10.1155/2014/317980

**Published:** 2014-06-11

**Authors:** Mathias Zink

**Affiliations:** Central Institute of Mental Health, Department of Psychiatry and Psychotherapy, Medical Faculty Mannheim, Heidelberg University, P.O. Box 12 21 20, 68072 Mannheim, Germany

## Abstract

Insight into the biological pathomechanism of a clinical syndrome facilitates the development of effective interventions. This paper applies this perspective to the important clinical problem of obsessive-compulsive symptoms (OCS) occurring during the lifetime diagnosis of schizophrenia. Up to 25% of schizophrenia patients suffer from OCS and about 12% fulfil the diagnostic criteria of obsessive-compulsive disorder (OCD). This is accompanied by marked subjective burden of disease, high levels of anxiety, depression and suicidality, increased neurocognitive impairment, less favourable levels of social and vocational functioning, and greater service utilization. Comorbid patients can be assigned to heterogeneous subgroups. It is assumed that second generation antipsychotics (SGAs), most importantly clozapine, might aggravate or even induce second-onset OCS. Several epidemiological and pharmacological arguments support this assumption. Specific genetic risk factors seem to dispose patients with schizophrenia to develop OCS and risk-conferring polymorphisms has been defined in *SLC1A1*, *BDNF*, *DLGAP3*, and GRIN2B and in interactions between these individual genes. Further research is needed with detailed characterization of large samples. In particular interactions between genetic risk constellations, pharmacological and psychosocial factors should be analysed. Results will further define homogeneous subgroups, which are in need for differential causative interventions. In clinical practise, schizophrenia patients should be carefully monitored for OCS, starting with at-risk mental states of psychosis and longitudinal follow-ups, hopefully leading to the development of multimodal therapeutic interventions.

## 1. Introduction

### 1.1. Insight into Biological Mechanisms of Diseases

#### 1.1.1. Mental Disorders Are a Major Cause of Disability

Clinical research in psychiatry has achieved some important progress both in pathogenetic concepts and in therapeutic interventions over the past decades. However, compared to other medical illnesses and disciplines biological mechanisms of psychiatric disorders are still poorly understood. This leads to a lack of innovative therapeutic interventions in psychiatry compared, for example, to general medicine [1]. Relating to schizophrenia, the market approval of first and second generation antipsychotics (FGA, SGA) has to be acknowledged as the last important and seminal innovation in treatment. Besides pharmacological interventions [2], cognitive behavioral therapy (CBT) is still scarcely implemented in the clinical management, although it is supported by convincing evidence and current treatment guideline recommends its use in schizophrenia [3–8]. As a consequence of missing treatment improvement, problems caused by stigmatization are still apparent today [9].

Differences in the degrees of insight into the biological mechanism of diseases lead to marked differences of effective treatment conditions in general medicine and in psychiatry. Compared to other chronic disorders such as hypertensive heart disease, diabetes mellitus, or even cancer, treatment of schizophrenia is confined to a small number of substances and pharmacological mechanisms. Noteworthy, the effect sizes achieved with core psychopharmacological agents are often in the range of medial and sometimes large improvements [10]. However, the last decades have shown that the time needed for innovative drug development and market approval is much longer in psychiatry [1].

Few examples from general medicine might elucidate how basic research of disease mechanisms facilitated the development of causative treatment: treatment of hypertension gained profit from insight into the regulation of blood pressure by the renin-angiotensin system. As a result of these findings entirely new substances could be developed. In diabetes, several additive mechanisms are currently used for treatment, starting with metformin, sulfonylurea, thiazolidinediones, glucagon-like receptor 1 antagonists, and different formulations of insulin. Finally, the multimodal treatment of cancer implemented antibody-based cytostatic substances based on molecular targets which had been defined in careful basic research.

Of course, in other medical areas, such as several subtypes of cancer, Huntington's disease, and subtypes of dementia the insight into the molecular mechanism did not yet result in causative treatment. In general, further research seems necessary in order to define molecular targets for interventions. This is especially true for major psychiatric conditions such as schizophrenia or obsessive-compulsive disorder (OCD) and even more for their comorbidity.

### 1.2. Pathogenic Concepts on Schizophrenia and Obsessive-Compulsive Disorder

Schizophrenia [11] is perceived as a common final clinical manifestation of several different and heterogenous neurobiological processes. The interaction of genetic and environmental factors is considered to be of critical importance. Genetic properties alter early neural development and elicit long-lasting effects due to persisting plastic processes of pre- and perinatal development [12–14]. In a neurochemical perspective, alterations of dopaminergic, serotonergic, and amino acid neurotransmission have been defined within the modified dopamine [15] or the glutamate hypothesis of schizophrenia [16, 17]. The final phenotypic manifestation of a psychotic episode occurs rather late in the disease progression of schizophrenia. It has been proposed that therapeutic interventions addressing the early stages of early events in disease progression might be even more effective than curing the late phenotypic stages, namely, the psychotic episode [18, 19]. This paradigmatic change has been propagated by Insel using the term of “rethinking schizophrenia” [20].

Current concepts of OCD localize the critical pathogenic processes within the fronto-striato-thalamocortical circuitry connecting the orbitofrontal cortex (OFC), the anterior cingulate cortex, and the basal ganglia (thalamus and caudate nucleus) [21–24]. Serotonergic neurotransmission seems to play a critical role, because treatment with serotonin-specific reuptake inhibitors (SSRIs) leads to symptomatic improvement and response to cognitive behavioural therapy accompanied by alterations of the brains serotonin system [25–29]. The disease risk is largely determined by genetic factors, but the only linkage finding that has been consistently replicated refers to single nucleotide polymorphism (SNP) in the gene* SLC1A1* on chromosome 9p24, encoding the neuronal glutamate transporter EAAC1 (excitatory amino acid carrier 1) [30]. This finding supports neurochemical concepts beyond the serotonergic theory of OCD. And indeed, independent lines of evidence support the glutamatergic theory of OCD, most importantly animal models [31–34], human MR spectroscopy [35, 36], treatment approaches addressing the glutamatergic system [37–42], and finally genetic studies [43–45].

### 1.3. Treatment Resistance

Due to limited insight into the biological mechanisms of both schizophrenia and OCD the modes of treatment are confined to few strategies. Consequently, a large proportion of patients do not sufficiently respond to treatment, even if clinicians follow guidelines for multimodal treatment approaches in schizophrenia [2, 46, 47] or in OCD [25, 48–51]. Faced with treatment resistant patients, pharmacological strategies of polypharmacy are often used in both schizophrenia [47] and OCD [52–54].

Therefore, it is not astonishing that the comorbidity of both syndromes challenges research approaches and treatment options even more. This review has the intention to summarize the current knowledge of the pathogenesis and therapeutic options of obsessive-compulsive symptoms (OCS) in schizophrenia patients and describes necessary future research perspectives.

## 2. Main Part

### 2.1. Obsessive-Compulsive Symptoms in Schizophrenia: Epidemiology

**Figure 1:**
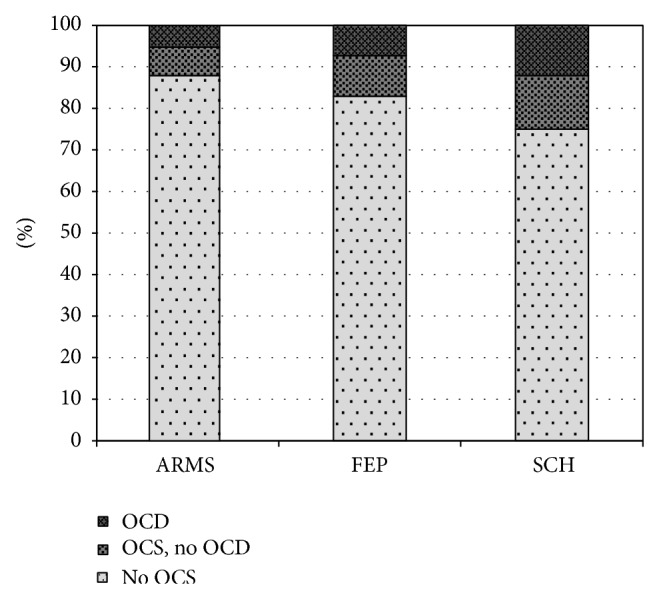
Estimations on prevalence of OCS and OCD according to different samples of patients. (1) Mean prevalence rates in at-risk mental state studies (ARMS). (2) Mean prevalence rates in first episode psychotic patients. (3) Mean prevalence rates in schizophrenia patients.

Patients with schizophrenia have a high lifetime risk for OCS of about 25% and recent meta-analyses concluded that at least 12% also fulfil the criteria for an OCD (Figure 1) [55–63]. In contrast, in the general population the prevalence rates for OCD are only 1 to 2% [64]. Patients suffering from primary OCD carry a relatively low risk (1.7%) to develop comorbid psychotic symptoms [65].

Schizophrenia patients, who suffer from comorbid OCS, often display pronounced psychotic and sometimes treatment resistant symptoms [66, 67]. In addition, specific neurocognitive deficits have been described [68]. Comorbid patients more often utilize health care services [69] and show heightened levels of anxiety and depression leading to increased risk for suicidality [59]. These pronounced impairments increase the burden of disease; they lead to poorer social and vocational function [70–73] and a less favourable overall prognosis [74].

### 2.2. Differentiation between OCS and Psychotic Symptoms

Psychotic symptoms and OCS can often be clearly distinguished, but sometimes a marked overlap between dimensions of schizophrenia and the obsessive-compulsive phenotype [75] makes careful differentiation and classification of presented symptoms necessary. The clinical exploration should focus on several aspects that help discriminate delusions or hallucinations from typical OCS to ensure valid and reliable diagnosis [76, 77]. Delusions are defined by the characteristics of certainty, incorrigibility, and impossibility or falsity of their content. The subjects believe in them with absolute conviction, despite compelling counterarguments or proof of the contrary. Thus, delusions describe implausible, bizarre, or patently untrue “facts.” Hallucinations are perceived with the character of sensory information originating from an external source. The subject classically attributes this thought content not to his own thinking. In contrast to these psychotic symptoms, obsessions and compulsions are intrusive thoughts/actions that originate from the subjects' own thinking. The patients report insight into the unreasonable nature and try to resist or ignore them. In clinical practice several aspects have to be kept in mind.

*The Criterion of Insight*. Patients suffering from OCD typically fulfil the abovementioned three symptom characteristics: they attribute the obsessions, impulsive symptoms, and compulsions to their own thinking, declare with insight their unreasonableness, and show some degree of resistance against them. The first characteristics allow a differentiation from hallucinations and delusions. Ruminations or stereotypic egodystonic cognitions with direct relation with the contents of psychotic thinking should not be diagnosed as obsessions. 

*Stereotypic Behaviour with Relations with the Psychotic Thought Content or Compulsions*. Cleaning or checking behaviour should only be diagnosed as compulsions if it is accompanied by typical obsessions and not, if the patient currently suffers from delusions of contamination, intoxication or infection. 

*Obsessions Presented as Pseudohallucinations*. A subgroup of OCS patients experiences their obsessions as extremely aversive and burdening. These patients may try to distance themselves by using expressions such as “voices” or “foreign thought content,” but in most cases these phenomena can be characterized as pseudohallucinations and differ on a phenomenological level from true hallucinations. 

*Reevaluation of OCS after Remission of Psychotic Symptoms*. OCS might manifest for the first time simultaneously with the first psychotic exacerbation. Here, the final decision whether the patient really suffers from a valid comorbid condition should be postponed until the remission of psychotic symptoms.

*Differentiation of OCS from Catatonic Symptoms.* Particularly catatonic schizophrenia [78] confers several problems to psychopathological assessment in daily clinical practice. Even the established psychometric scales such as the catatonia rating scale [79] and the Yale-Brown-Obsessive-Compulsive Scale [80, 81] share many symptomatic dimensions. Historically, a more precise characterization and differentiation of symptoms was achieved by an undisguised view on the natural long-term course of schizophrenia. The work of Karl Leonhard [82] provided case descriptions that allowed a clear discrimination between OCS and catatonic symptoms most importantly in patients with the so-called “manieristic catatonia.”

### 2.3. Homogeneous Subgroups within the Large Comorbid Sample

The astonishingly large cohort of schizophrenia patients with OCS has been subdivided into several homogeneous subgroups depending on the diverse clinical course and phenotypic presentation. It appears to be necessary to focus on these subgroups with common clinical properties in order to unravel the specific interplay of genetic, psychosocial, and pharmacological factors. The subdivision into such subgroups can be guided by rather simple clinical criteria, such as the time point of first manifestation of comorbid OCS and the clinical course over time.

#### 2.3.1. First Manifestation of OCS

The onset of OCS has been described at different stages during the course of the psychotic disorder:

1. before psychosis as an independent, coexisting syndrome and diagnosed as OCD;
2. prior to psychotic manifestation as part of the at-risk mental state (ARMS);
3. in parallel to the first manifestation of psychosis;
4. during the course of chronic schizophrenia;
5. as markedly aggravated or second-onset (*de novo*) OCS after initiation of antipsychotic treatment.

**Figure 2:**
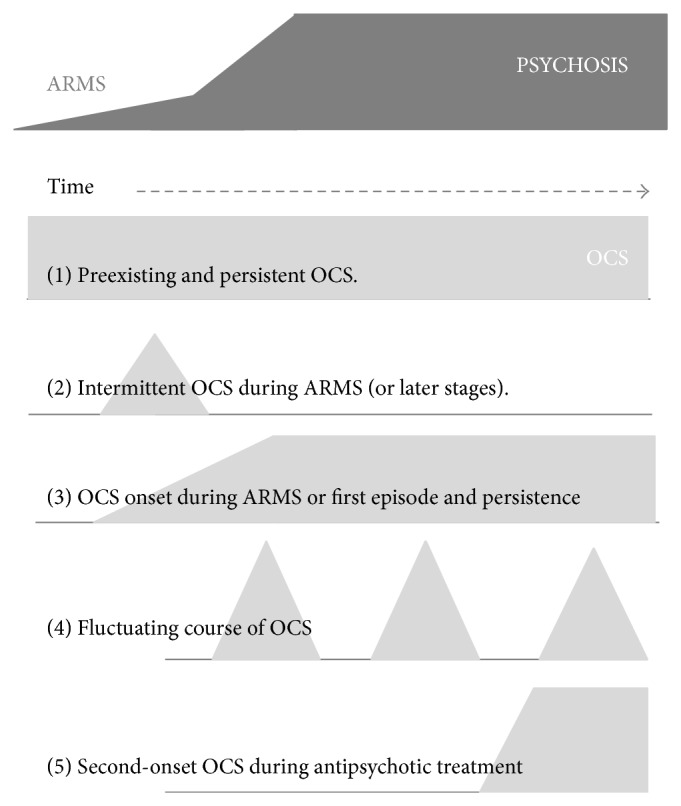
Schematic diagram on onset and course of OCS related to stages of schizophrenia. Bright-gray symbols indicate the onset and severity of OCS, dark-gray symbols are related to the at-risk mental state of psychosis (ARMS), the upcoming first episode of schizophrenia or its chronic course. (1) Preexisting and persistent OCS. (2) Intermittent OCS during ARMS or later in the clinical course. (3) OCS onset during ARMS and persistent course, strongly associated with the psychotic symptoms (schizo-obsessive concept). (4) Fluctuating course of OCS. (5) Second-onset OCS during antipsychotic treatment.

A remarkably large subgroup of patients already suffers from OCS during the ARMS, as recently summarized [83]. The mean prevalence of all reported sample-size weighed rates results in 12.1% (CI: 9.4 to 14.8%) of ARMS patients who report OCS [84–88], whereas 5.2% (CI: 4.1 to 6.3%) fulfil the criteria for OCD [84, 86–92] (Figure 1). In first episode patients slightly higher averaged rates can be found for OCS (17.1%, CI: 14.0 to 20.2) and OCD (7.3%, CI: 5.3 to 9.3%) (Figure 1) [70, 83, 88, 88, 93–95]. Epidemiological data in the referred individual studies largely vary. This might be explained by differences in the ARMS criteria used and differences in the definition and psychometric assessment of OCS or OCD. OCS during the ARMS seems to have an important impact on other clinical variables, but so far findings have been rather heterogeneous. Consistent results have been reported for higher impairment of psychosocial functioning [85, 89, 91, 94] and more severe depressive symptoms [70, 86, 89, 96] in cases with comorbid OCS. In contrast, results investigating the effect of OCS on the transition rates into psychosis [86, 90, 91, 96] have been contradicting. Only preliminary data existed regarding the influence on cognition [85, 96, 97], until the interventional study PREVENT (Secondary Prevention of Schizophrenia: A Randomized Controlled Trial [84]) allowed a multidimensional assessment of a large cohort [83]. Within a sample of 233 ARMS patients 26 patients fulfilled the DSM-IV criteria for concurrent OCD or had a lifetime history of at least subclinical OCS. They were more severely impaired in psychosocial functioning and general psychopathology but not regarding affective symptoms and neurocognitive abilities. Apart from OCS during the ARMS several studies investigated the cooccurrence of OCS during manifest schizophrenia. Whereas some patients experience OCS onset simultaneously with the first episode of psychosis, another and often underestimated subgroup reports OCS development after treatment-start with SGAs. In these cases, a typical order of three events can be observed: first “onset of psychosis,” second “start with SGA treatment,” and subsequently “*de novo *development of OCS.” This sequence suggests the involvement of pharmacodynamic mechanisms in the pathogenesis of OCS in this subgroup of schizophrenia patients (see Figure 2(5) and detailed description in Section 2.6).

#### 2.3.2. Clinical Course of OCS over Time

Not only do the time points of first manifestation of OCS differ between subgroups of comorbid patients, but also the longitudinal course of symptom severity differs (Figure 2). OCS may present as fluctuating symptoms; they may resolve, persist, or even worsen over time. Within patients who reported manifest OCD prior to the psychotic illness, for example, adolescents, OCS most likely persisted or worsened independently of the course of schizophrenia [98]. Only few longitudinal studies investigated quantitative changes of OCS severity within the course of schizophrenia. One large investigation from the Netherlands evaluated participants over a period of 5 years and described a predominantly fluctuating course of OCS severity in over 70% of the comorbid sample: some patients experienced the remission of OCS and others experienced a fluctuating, more or less cyclic course, more or less cyclic course. Smaller groups reported first onset of OCS or persisting symptom severity [70]. A second longitudinal study in a German sample investigated schizophrenia patients during treatment with different modes of antipsychotic monotherapy. Schirmbeck et al. found persisting OCS severity over 12 months in the group treated with clozapine (CLZ) and olanzapine (OLZ) in contrast to low comorbidity rates in the group treated with amisulpride (AMS) or aripiprazole (APZ) [68, 99].

In conclusion, the diverse clinical course adds to the heterogeneous clinical presentation and suggests an involvement of different aetiological factors. The influence of environmental factors and/or interactions of psychopathological symptoms on the longitudinal development of comorbid OCS in schizophrenia can be assumed but needs further investigation (see Section 3.1.2).

### 2.4. Heterogeneous Pathogenic Concepts

On the basis of different time points of first onset and different clinical courses over time, several pathogenetic concepts have been proposed. The proposed theories often overlap in important aspects but sometimes also contradict each other.

The two rather common psychiatric syndromes could of course manifest together by chance, representing a random association. However, based on the abovementioned high prevalence rates and the highly diverse clinical presentations this cannot be the only explanation for OCS in nearly every fourth patient with schizophrenia.

In general, nosological concepts of OCD substantially changed during the history of psychopathology. French and German pioneers of psychiatry published very divergent theories in the 19th and 20th century [100]. The relationship between OCD and the delusional spectrum and the so-called unitary psychosis was a major matter of discussion. Some authors assumed that patients with schizophrenia might develop OCS as an attempt to reduce psychotic symptoms. Thus, the presence of OCS was proposed to have protective effects regarding psychotic disintegration. These explanations were based on single-case analyses or small case series [101, 102]. Quite in line with these statements, Guillem et al., who applied reliable methods of psychopathology and epidemiology, described negative correlations between specific OCS and the severity of psychotic disorganization in thinking and behaviour and proposed compensating mechanisms [103]. In a broader perspective, subsequent research revealed the abovementioned negative impact of comorbid OCS on general severity of illness; for example, a higher severity of psychotic symptoms and more functional impairment of OCS were present [66] (see above).

The cooccurrence of the syndromes might be approached from both the OCD and the schizophrenia spectrum, resulting in different semantic and nosological concepts. In the OCD spectrum perspective, the concept of “schizotypic OCD” has been described [104, 105]. Here authors assume a complex association of primary OCD with schizotypal personality disorder, which meets DSM related criteria. This subgroup of primary OCD patients presents beliefs, which can be classified on a spectrum between obsessions and delusions [100]. They are emphasizing the similarities as being irrational thoughts, the first with insight and the latter lacking insight. Quite similarly, the category of “obsessions without insight” has been integrated into the fourth edition of the Diagnostic and Statistical Manual (DSM IV). It has been hypothesized that OCD patients without insight might represent a subgroup with genetic, phenotypic, and therapeutic vicinity to the schizophrenia-like spectrum [106, 107].

Approaching the cooccurrence from the schizophrenia spectrum, Poyurovsky et al. proposed the so-called “schizo-obsessive” subtype of psychosis, based on careful cross-sectional evaluations [108]. Patients with this subtype are thought to suffer from OCS in addition to positive, negative, and cognitive schizophrenia symptoms [62]. Hwang et al. [109], Bottas et al. [110], and Reznik et al. [111, 112] published similar concepts. So far, the attempts to validate the “schizo-obsessive” subtype on a neurobiological level have been inconsistent. Some but by far not all studies were able to describe specific neurological features [113, 114], cognitive deficits [115, 116], and even structural abnormalities [117].

Finally, the mentioned high prevalence rates of OCS during the ARMS led to the assumption that specific OCS could be a part of the ARMS, in particular the basic symptom cluster in the early course of schizophrenia [118, 119].

The summarized pathogenic concepts reflect the high degree of heterogeneity within the comorbid sample. At present, the number of publications on this topic nearly doubles every year. The main attempt of current research is to elucidate pathogenic mechanisms in order to better understand the diversity in time of onset, clinical course over time, and pathogenic concepts.

### 2.5. Underlying Neurobiological Mechanisms and Environmental Factors

Whereas the above described explanatory concepts mainly follow a clinical or psychopathological rationale, several investigations focus on a neurobiological perspective. So far, most emphasis has been given to neuropsychology, namely, a multimodal neurocognitive characterization. Preliminary investigations of neurological soft signs [113, 114] and neuroimaging techniques [117, 120] need replication.

#### 2.5.1. Neurocognitive Correlates of OCS in Schizophrenia

In primary OCD specific neurocognitive deficits have consistently been replicated. Especially cognitive shifting abilities, inhibitory control, and the application of effective planning strategies have been described as core cognitive domains [121]. Therefore, the question arose whether OCS in schizophrenia might also be linked to additional cognitive impairment in these OCD-related domains [59]. Several authors tried to differentiate schizophrenia samples with versus without comorbid OCS regarding neuropsychological performance with partially contradicting results. Whereas several authors in some cases small investigations did not find any significant differences [55, 73, 107, 122–126], and others even suggested that OCS may be associated with better cognitive abilities [127, 128], especially in the prodromal states of schizophrenia [83, 85, 96, 97]. Most authors, however, showed more pronounced deficits in the described domains of executive functioning [109, 115, 116, 129], cognitive flexibility [130, 131], and also delayed visual memory [132, 133].

In his recent longitudinal assessment, Lysaker et al. analysed executive functioning and reported that deficits were linked to greater concurrent and prospective self-report of OCS among schizophrenia patients [115]. Our comprehensive and prospective investigation explicitly included OCD-related cognitive domains [121, 134]. Over a period of 12 months we observed that schizophrenia patients with comorbid OCS showed significant pronounced deficits with increasing effect sizes with respect to cognitive flexibility, visuospatial perception, and visual memory. In addition, performance in these domains correlated with OCS severity [68].

These neuropsychological findings have been proposed to reflect possible causal pathways. Here, it has been assumed that pronounced cognitive deficits may reflect an underlying neurobiological risk factor for schizophrenia patients to develop OCS. Within this perspective, at least partially overlapping neurobiological mechanisms with OCD have been assumed. In order to further substantiate this hypothesis neurobiological mechanisms, which might explain the pronounced deficits in the comorbid sample, should be defined. Therefore, pathogenic research should focus on candidate brain regions, which have been described in primary OCD, such as increased activation levels in the orbitofrontal cortex [135, 136] using functional magnetic resonance imaging (fMRI) approaches.

#### 2.5.2. Functional Magnetic Resonance Imaging

Both OCD and schizophrenia patients were thoroughly investigated with different neuroimaging methods, most importantly structural and functional magnetic resonance imaging (fMRI) [135, 137, 138]. Alterations in partly overlapping brain regions were described, but the differences in disease-specific changes are out of the focus of this review. So far, only four neuroimaging studies investigated the neural correlates of OCS in schizophrenia. In a structural approach Aoyama et al. reported a significant volume reduction of the left hippocampus in schizophrenia patients with OCS [139]. Levine et al. found negative associations between OCS and the activation of the left dorsolateral prefrontal cortex during a verbal fluency task [140]. Finally, Bleich-Cohen et al. compared groups in a working memory paradigm. Independently of additional comorbid OCS schizophrenia patients performed worse and showed less activation in dorsolateral prefrontal cortex and right caudate nucleus, when compared to healthy controls [141]. Noteworthy, recruitment in these studies was solely based on the clinical phenotype not accounting for possible underlying pharmacodynamic aspects. Furthermore, no study particularly assessed the fronto-striato-thalamocortical circuitry connecting OFC, anterior cingulate cortex, thalamus, and caudate nucleus, although these regions are thought to play a core role in the pathogenesis of OCD [21, 22, 27].

In our recent investigation of neural correlates of SGA-induced OCS in schizophrenia, we stratified patients according to their antipsychotic monotherapy into two groups ((I) CLZ or OLZ; *n* = 21; (II) AMS or APZ; *n* = 19) and applied a Go/NoGo task assessing inhibitory control and an *n*-back task measuring working memory.

Patients of group (I) showed significantly more severe OCS and pronounced impairments in specific neurocognitive abilities. Brain activation patterns did not differ during the working memory task, but group (I) patients showed significantly increased activation in the OFC during response inhibition. These alterations in OFC activation were significantly associated with the severity of reported obsessions and impairment in specific neurocognitive tasks [120]. Further longitudinal research seems to be necessary in order to define the neural correlates of an increased risk for OCS, for specific pharmacological side effects (see below) and for specific subtypes of obsessions and compulsions.

#### 2.5.3. Neurotransmitter Systems

Additional neurobiological mechanisms with an impact on OCS have to be acknowledged within the neurochemically defined neurotransmitter systems. Current pathogenic theories of OCD assume a central serotonergic dysfunction in the mentioned network comprising cortical, striatal, and thalamic centres [23]. Therapeutic effects of selective serotonin reuptake inhibitors (SSRIs) and cognitive behavioural therapy (CBT) on serotonergic neurotransmission in this region support this assumption [28, 29]. These findings led to the assumption that the strong serotonergic antagonism of CLZ [142–144] and OLZ [145] might constitute a pathogenic mechanism in the development of second-onset OCS in schizophrenia (for more details see Section 2.6). However, other neurotransmitter systems also have to be considered. Alterations in dopaminergic activity [146] and in glutamatergic neurotransmission have been related to OCD: support for the involvement of glutamate in the development of OCD [38] comes from animal models [31, 32, 34], human MR spectroscopy [35, 36], treatment approaches addressing the glutamatergic system [37, 39–42], and genetic studies.

#### 2.5.4. Genetic Disposition

While schizophrenia and OCD are common psychiatric disorders with strong heritability [44, 45, 147, 148], the results of family and molecular studies of both disorders do not show much overlap. Regarding OCD genetic association studies on candidate genes of serotonergic and dopaminergic neurotransmission were rather ambiguous. So far, the only linkage finding, which has been consistently replicated, refers to single nucleotide polymorphism (SNP) in the gene* SLC1A1* (solute carrier family) on chromosome 9p24, encoding the neuronal glutamate transporter EAAC1 (excitatory amino acid carrier 1) [30, 149–153]. Recently, Porton et al. reported alternative splicing of SLC1A1 [33]. Three evolutionary conserved and widely expressed isoforms modulate or even inhibit glutamate uptake.

A possible genetic disposition to comorbid OCS in schizophrenia has just recently become a focus of interest (see Section 2.6.3). So far, methodological concerns such as the restriction to mainly cross-sectional evaluations and a lack of power due to small sample sizes added to inconclusive findings. Thus, progress in pathogenic understanding seems most likely if future research focuses on the multimodal characterization of homogeneous subsamples, for example, patients who develop secondary OCS during SGA treatment.

This next section summarizes evidence supporting this hypothesis by reporting epidemiological and pharmacological arguments as well as genetic findings.

### 2.6. OCS Induced by Second Generation Antipsychotics

Some patients report the mentioned schematic order of first onset or aggravation of OCS after psychotic manifestation and treatment initiation with SGAs. Thus, simple clinical exploration helps identify this homogeneous group of patients. It is important to assess the time points of onset of the first psychotic manifestation, the start of antipsychotic treatment, and the subsequent onset of OCS [99, 154, 155]. During treatment with first generation antipsychotics (FGA) this pattern has rarely been observed. Several authors proposed a pharmacodynamic mechanism and attributed OCS to the important feature of balanced antidopaminergic and antiserotonergic properties of SGAs, in contrast to the low affinity of first generation antipsychotics to serotonergic receptors [156, 157]. In addition, differential effects of FGAs and SGAs on GABAergic and glutamatergic neurotransmission have to be considered [158, 159].

The first reports on OCS as a possible side effect of SGAs [154, 160] were published by Baker et al. [161] and De Haan et al. [162]. Since then several studies showed a clear association between SGA treatment, most importantly CLZ [155], and the* de novo* occurrence of OCS [93, 133, 160, 163]. Causal interactions have been proposed resulting in the expression of SGA-induced OCS [154, 160].

Before describing arguments supporting a causal association between CLZ treatment and OCS development, the general significance of CLZ needs to be mentioned. Without a doubt, this SGA must be considered a highly effective and indispensable part of the antipsychotic armament [144, 164–166], especially in cases with otherwise treatment resistant psychoses [167]. Not only the CATIE study [168], but also several other studies have demonstrated its superior antipsychotic efficacy [169–172]. Therefore, CLZ is the antipsychotic of first choice in treatment of resistant schizophrenia. In addition, treatment with CLZ exerts important antisuicidal effects resulting in low mortality rates of CLZ-treated schizophrenia patients [173]. However, important metabolic and haematological side effects have to be kept in mind [169] and related to the topic of this paper the* de novo* occurrence or exacerbation of OCS under antipsychotic treatment has most often been observed with CLZ [154, 155, 163]. In order to formally prove this hypothesis, a randomized controlled trial would be necessary, according to the general criteria suggested by Hill [174]. However, due to legal and ethical restriction such a trial cannot easily be performed. Nevertheless, several epidemiological and pharmacological arguments support this assumption (for summary see Table 1).

#### 2.6.1. Epidemiological Evidence

*The Prevalence of OCS Increased after Market Approval of SGAs*. The cooccurrence of OCS did not gain much clinical awareness, while treatment with different FGAs was the first-line therapy in schizophrenia. Only few investigations reported comorbidity rates in these samples [69, 71, 175, 176]. The situation markedly changed, when SGAs were formally approved for the treatment of schizophrenia, most importantly CLZ, in the 1970s in Europe and the late 1980s in the USA [166, 177]. After this paradigmatic change in treatment, the prevalence estimations markedly increased. Of course, a potential publication bias and increased general awareness of this topic over time have to be considered. Nevertheless these data provided a first and indirect hint towards a possible interrelation.

*Increased OCS Prevalence in Later Stages of Schizophrenia*. Compared to ARMS and first episode samples, the prevalence estimations of OCS and OCD in manifest schizophrenia are significantly higher (12% (OCD) and 25% (OCS); see Figure 1). The higher rates in the later stages of the disease might partly be attributed to antipsychotic treatment with proobsessive SGAs.

*Onset of De Novo OCS or Marked Aggravation during Antipsychotic Treatment*. In several case reports and cases series, as well as systematic evaluations, the* de novo* emergence of OCS during the treatment with atypical antipsychotics, most importantly CLZ [155], has been described. Although most of these reports are limited to a simple narrative design, the number of independent observations supports the assumption of a causal interrelation.

*High Prevalence Rates during CLZ Treatment and Associations Suggesting OCS Induction by SGAs*. Impressively high proportions of comorbidity rates have been attributed to the pathogenic process of SGA-induced OCS by several authors. Poyurovski et al. estimated that up to 70% of schizophrenia patients treated with proobsessive SGAs develop secondary OCS [61], while Lykouras et al. reviewed published data and reported* de novo* OCS in 77% of CLZ-treated patients [154]. Further independent studies reported even higher numbers of SGA-induced OCS within their samples of comorbid patients, ranging from 29 of 39 (74%) [178] and 23 of 26 (88%) [179] to 25 of 28 patients (89%) [133]. Furthermore, retrospective assessments of the abovementioned three critical events reveal that most patients experience the onset of OCS after first manifestation of psychosis and the start with SGA treatment [180].

#### 2.6.2. Pharmacological Evidence

Further pharmacological evidence contributes to the assumption of proobsessive SGA effects.

*CLZ-Treated Patients Suffer from OCS More Often*. When comparing patients according to their mode of antipsychotic treatment, the risk for comorbid OCS markedly differs. As reported, high prevalence rates in CLZ-treated patients [181] contrast with low rates during treatment with FGAs, for instance, haloperidol [67] or other SGAs. These diverging findings might be explained by the mentioned differences in pharmacodynamic properties, in particular regarding inherent serotonergic blockade, monoaminergic reuptake inhibition, or even partial serotonergic agonism [143, 158, 182–184]. In contrast to the high numbers of comorbid OCS during CLZ treatment, APZ, a partial dopaminergic and serotonergic agonist, was associated with an inherent antiobsessive effect in schizophrenia patients with OCS [185–189], quite similar to AMS, a dopamine D3/D2 receptor antagonist [190, 191].

In line with these results we found comorbid OCS in more than 70% of patients treated with CLZ or OLZ, whereas less than 10% of patients treated with AMS or APZ reported OCS [133]. Vice versa, grouping schizophrenia patients according to presence or absence of comorbid OCS revealed that 77% of comorbid patients were treated with CLZ, whereas only 36% of those without OCS received this substance [179]. Although these results clearly suggest an association between CLZ treatment and comorbid OCS, a possible confounding effect due to the selection of specific SGAs for specific subgroups of patients has to be considered.

*Associations between Pharmacological Variables and OCS Severity*. Recent research indicated a dose-effect relation. The severity of OCS was found to be positively correlated with duration, dosage, and serum levels of CLZ treatment.

*Duration of Treatment*. Lin et al. [178] compared CLZ-treated patients with and without comorbid OCS and found significantly longer CLZ treatment periods for the comorbid group but no difference in duration of illness. Accordingly, Schirmbeck et al. reported a positive association between OCS severity and duration of CLZ treatment [133] and de Haan et al. reported this association for OLZ [192].

*Dosage and Blood Serum Levels*. Similar to association with a longer treatment duration, several authors demonstrated positive correlations with dose or serum levels of CLZ [60, 133, 163, 178]. Furthermore, the reduction of daily CLZ dosage, for instance, through the combinations with another SGA, such as APZ, resulted in an alleviation of OCS severity [187, 189, 193]. Noteworthy, this observation might represent both a reduction of the suggested dose-related side effect of CLZ and a consequence of inherent antiobsessive effects of APZ. The latter assumption was supported by a* placebo* controlled randomized trial, which showed reduced OCS severity after combination with APZ but unchanged CLZ dose during the course of the study [185].

*The Longitudinal Course of OCS during Treatment with Different SGAs*. A recent longitudinal study revealed differential effects of SGAs on the course of comorbid OCS. Whereas a CLZ/OLZ group showed persistently high OCS severity over a 12-month observational period, AMS/APZ group reported decrease of the initially already low symptom severity. These divergent changes resulted in significant differences between the two pharmacologically diverse groups (completer analysis: *P* = 0.006; full sample analysis: *P* = 0.007) [99].

Noteworthy, contradicting findings regarding the treatment with CLZ have been reported, where the addition of CLZ [194], an increase in CLZ dosage [154], or start with OLZ treatment [108, 195] has been associated with an alleviation of OCS severity. One explanation for these heterogeneous findings relates to the abovementioned diagnostic difficulties to differentiate between OCS and delusional or catatonic symptoms. Patients, who show obsessive ruminations or stereotypic thoughts during acute psychosis or repetitive ritualized behaviour clearly related to the patient's primary psychotic condition might indeed benefit from treatment with CLZ. Furthermore, positive effects of antipsychotics have also been reported in primary OCD, including OLZ, especially in cases with treatment resistance to serotonergic antidepressants [25, 52, 53, 196]. Nevertheless, even in treatment resistant OCD current treatment guidelines do not recommend CLZ as an augmentation strategy. It has been proposed that OCS during CLZ treatment might differ from symptoms in primary OCD [76], but assessments in large samples are missing.

In summary, reported evidence strongly suggests an association between comorbid OCS in schizophrenia and SGA treatment, in particular with CLZ. The published epidemiological and pharmacological evidence hints at causal interactions, suggesting that CLZ's strong inherent antiserotonergic properties [164, 166, 197], most importantly the antagonism at 5-HT1C, 5-HT2A, and 5-HT2C receptors [142, 143, 198], represent an relevant pathogenic mechanism. Low affinities to dopamine receptors result in a very small ratio of dopaminergic/serotonergic receptor blockade, which largely differs from other SGAs such as AMS or APZ [184, 199, 200]. Reciprocal interactions of dopaminergic and serotonergic neurotransmission with glutamatergic and GABAergic functions also need to be considered [158].

Within a broader perspective on SGA-induced OCS, additional questions arise concerning predisposing factors for OCS in schizophrenia. These might comprise external factors such as psychosocial stressors and patient-inherent characteristics (neurocognitive profile, the subtype of psychosis, the stage of the illness, any kind of affective comorbidity, or a family history for anxiety disorders). Not least, the individual genetic disposition seems to be highly important.

#### 2.6.3. Genetic Disposition to Comorbid OCS in Schizophrenia

Based on the replicated associations with the gene* SLC1A1* in primary OCD patients, a South Korean research group investigated the genetic risk to develop second-onset OCS during treatment with SGAs [160]. Analyses investigating associations between specific SNPs of the candidate gene* SLC1A1 *and SGA-induced OCS in this sample showed strong associations with the A/C/G dominant haplotype rs2228622/rs3780413/rs37801412. With an odds ratio of 3.96 the likelihood for patients who carried this A/C/G haplotype was almost 4 times higher to suffer from SGA-induced OCS. A replication approach in 103 schizophrenia patients of European descent could not reproduce these findings either in single marker analyses, or in haplotype analyses. Nonsignificant results and considerably smaller odds rations suggested a lack of power and the necessity that investigations in much larger samples are needed [180].

In a subsequent study, Ryu et al. further described a genetic interaction of the* SLC1A1* polymorphism with variants in the gene* DLGAP3* (disks large associated protein 3) and a link to SGA-induced OCS [201]. Another study of a Chinese sample reported an interaction of SNPs in SLC1A1 and the type 2B subunit of the N-methyl-D-aspartate receptor gene (GRIN2B), as well as significant interactions with OCS severity [202]. Finally, based on associations between the Val66Met polymorphism and OCS in schizophrenia, the brain derived neurotrophic factor (*BDNF*) has recently been proposed as a forth candidate gene [203]. So far, independent replication approaches regarding* BDNF*,* DLGAP3*, and GRIN2B have not been conducted and further studies are needed to untangle the interplay of pharmacological and genetic risk factors for OCS in schizophrenia [180, 204].

The summarized evidence from epidemiological, pharmacological, and genetic studies proves that pharmacotherapy constitutes a relevant environmental factor, which might exert proobsessive effects in schizophrenia patients. Recently, Doyle et al. [205] tried to differentiate CLZ-associated OCS from OCD symptoms in a phenotypic approach. The authors observed in their small samples prevalent “doubting” in CLZ-treated patients, while OCD patients presented predominantly with the behavioural symptom of washing.

## 3. Discussion of Research Perspectives 

### 3.1. Gene and Environment Interactions

Several frequent and disabling mental disorders manifest as a consequence of both genetic and environmental factors. Schizophrenia, for instance, is commonly perceived as a result of gene and environment interactions (GxEIs), where individual genetic properties dispose to a specific liability and sensitivity for specific stressors. These could include migration to an urban surrounding, other stressful life events, or effects of psychotropic substances [11, 148, 206]. Similar concepts were suggested regarding depression [207], anxiety disorders [208, 209], and obsessive-compulsive disorder [44, 45]. Extending this perspective to common comorbidities it is even more complex and demanding to investigate whether these can also be described on the basis of GxEIs. One example has been illustrated by the described investigation of the risk to develop secondary OCS during treatment with SGAs.

#### 3.1.1. Treatment with Proobsessive SGAs

The above summarized evidence suggests that SGAs increase the risk for secondary OCS via a pharmacodynamic mechanism and thereby represent a relevant environmental factor. Independently, a set of genetic risk constellations, particularly within the gene* SLC1A1*, seem to predispose to OCS. However, the failure to replicate the initial results in the Republic of Korea sample [160, 180] suggests that the general genetic background of a patient (Asian or European) might be of importance when a specific SGA (balance between dopaminergic and serotonergic blockade) is introduced as the treatment of choice. Furthermore, gene-x-gene interactions (SNPs in* SLC1A1*,* BDNF*, DLGAP3, and GRIN2B) also seem to play a role [201, 203] and should be considered in forthcoming studies. It is an important progress in recent neurobiological research to investigate how the interaction of these factors might influence the propensity of schizophrenia patients to suffer from comorbid OCS when being treated with SGAs.

#### 3.1.2. Psychosocial Stressors

In addition, further nonpharmacological environmental factors should be investigated. Such factors might include psychosocial stress induced by critical life events, interpersonal factors, changes of the vocational situation, or the present state of general physical health. Fluctuation of OCS severity in the majority of first episode patients who have been followed up over 5 years [70] strongly suggests that these factors might have an effect on the severity and course of comorbid OCS. In addition, the reciprocal interaction and possible causal directions between OCS and psychotic positive, negative, and cognitive symptoms of schizophrenia must be unravelled and considered.

Detailed follow-up analyses are therefore needed. Patients, who recently reported changes in their OCS, should be investigated by means of an “Experience Sampling Method” (ESM). This approach captures the reactivity to environmental factors and the course of symptoms in detail on a day to day basis, in real life situations. Collected data will help identify the time course of symptom changes and its relation with important contextual triggers of variability.

Within this context it will also be desirable to collect DNA samples in order to analyse predisposing effects of the abovementioned polymorphism and to elucidate an increased risk for the development or aggravation of OCS after being exposed to stressful life events. Thus, combining experience sampling and genetic characterizations might markedly improve our insight into GxEI.

In conclusion, future progress might depend on two aspects. First, well defined homogeneous clinical cohorts should be defined to reduce the number of possible confounding causal factors to a minimum. The investigation of the order of symptom onset, the clinical course, pharmacological treatment, and further environmental factors should be integrated into prospective studies. Second, much larger cohorts have to be recruited in multicenter studies to investigate possible genetic risk constellations. Based on much smaller genetic risk estimations in the European sample [180], power analyses would result in group size calculations of about five thousand participants, which would be necessary for replication.

### 3.2. Treatment Approaches of OCS in Schizophrenia

As proposed in the introduction, insight into the biological pathomechanisms and effects of relevant environmental variables on the development and course of OCS in schizophrenia facilitates the development of effective treatment interventions.

#### 3.2.1. Pharmacotherapy

Based on our current knowledge, pharmacological combination and augmentation strategies have been suggested to improve comorbid OCS according to timely reviews [74, 108]. To address possible proobsessive effects of predominantly antiserotonergic SGAs, the add-on of mainly dopaminergic SGAs such as AMS and APZ has been proposed [186–190, 196, 210, 211]. Within augmentation approaches, the treatment with serotonergic antidepressants has been evaluated, for example, with the tricyclic antidepressant clomipramine [175] or with the SSRI fluvoxamine [98, 212, 213]. Results of these trials have been inconsistent with some studies reporting significant reduction of OCS, whereas others failed to observe this intended effect. Noteworthy, additive anticholinergic side effects and pharmacokinetic interactions have to be considered when combining substances. Finally, preliminary findings suggest promising results when augmenting with mood stabilizers such as valproic acid [214, 215] or lamotrigine [41, 216] (see Table 2).

#### 3.2.2. Cognitive Behavioral Therapy

As mentioned in Section 3.1.2, results from ESM studies could provide important information for individualised interventions, including adjusted modules of cognitive behavioural therapy (CBT). However, so far, very limited data exists on the efficacy and safety of CBT for schizophrenia patients with OCS. A recent review of published case reports and case series summarized data of 30 comorbid patients, who were treated with CBT including exposure elements or just exposure and response prevention alone [7]. Results showed favourable outcome measures with significant reduction of OCD severity in 24 patients. Within the included case series by Tundo et al. [217] over 50% of individuals who received CBT were classified as “much or very much” improved. Despite adverse clinical outcomes in 10% and a total dropout rate of 20%, preliminary results suggest meaningful and marked reduction of OCS severity in 80% of participants [74] (see Table 2).

In conclusion, the available evidence is certainly limited by the small case numbers.

Although CBT including exposure and response prevention is considered treatment of first choice for primary OCD with remarkably high effect sizes [48–51], with one exception, currently available CBT manuals for OCD do not provide guidelines for the treatment of OCS in schizophrenia [218–221]. Thus, further controlled clinical trials are certainly needed.

## 4. Conclusions

The summarized data substantiate the conclusions that OCS is a very frequent and relevant comorbid burden in schizophrenia. The clinical presentation of the cooccurrence is very diverse, suggesting different subgroups with heterogeneous pathogenic mechanisms. First insight into GxEI has been achieved for the subgroup of patients who experienced second-onset OCS during treatment with SGAs. First insight into biological mechanism of the pathogenesis of the comorbid condition has been achieved and creates the bases for the development of innovative therapeutic intervention. Further research should expand to different subgroups of the comorbid sample integrating additional factors which will most likely have an effect on the development and course of OCS. In particular, the effects of environmental stressors and their interaction with genetic properties are incompletely understood. In perspective, a broader set of environmental and genetic variables will have to be analysed in a longitudinal fashion, starting in the ARMS. This will not only improve the characterization of parallel subgroups, but also enhance the risk prediction regarding comorbid OCS. On an individual level risk factors could be assessed aiming at an early recognition and monitoring of emerging symptoms. The definition of an individual framework of predisposing and disease-provoking factors would further have an immediate impact on the application of therapeutic interventions, including both pharmacological and CBT approaches.

## Figures and Tables

**Table 1:** SGAs induce or aggravate OCS.

Clinical observations and epidemiological arguments	
(I)	The prevalence rates of OCS in schizophrenia increased after market approval of SGAs such as clozapine.
(II)	The comorbidity rates in later stages of schizophrenia are higher than during the ARMS or at first manifestation of psychosis.
(III)	In parallel to antipsychotic treatment OCS manifest *de novo *or show a marked aggravation.
(IV)	High prevalence rates of OCS are observed during CLZ treatment.
(V)	Schizophrenia patients with SGA-induced OCS markedly contribute to the entire sample of comorbid patients.

Evidence derived from pharmacological considerations	

(I)	Pharmacodynamic properties modulate the risk for OCS: marked difference between samples treated with first generation antipsychotics or mainly dopaminergic SGAs (such as aripiprazole or amisulpride) compared to CLZ.
(II)	OCS manifest as an unfavourable drug effect *de novo* during treatment with potent antiserotonergic SGAs such as CLZ.
(III)	Indicators of a dose-effect relation: the severity of OCS is positively correlated with duration, dosage, and serum levels of CLZ treatment.
(IV)	OCS severity persists over time in patients under stable CLZ treatment.
(V)	The severity of OCS improves after reduction of CLZ dosage to minimally sufficient levels (due to augmentation or combination).

Summary of epidemiological and pharmacological arguments supporting the induction or at least marked aggravation of OCS by SGA treatment as an unfavourable side effect. CLZ: clozapine, OCS: obsessive-compulsive symptoms, and SGA: second generation antipsychotic agents.

**Table 2:** Therapeutic interventions addressing OCS in schizophrenia.

Early recognition and monitoring	
(I)	Definition of at-risk constellations
(II)	Detection of subclinical levels of OCS or beginning cognitive impairment using sensitive sets of neurocognitive tests
(III)	Monitoring of apparent OCS

Add-on of psychotropic agents: polypharmacy	

(I)	Augmentation with antidepressants: clomipramine, fluvoxamine, and other SSRIs [level of evidence: RCTs, CS, CR] *Caveat*: additive (anticholinergic) side effects and pharmacokinetic interactions
(II)	Augmentation with mood stabilizers (lamotrigine, valproic acid) aiming at a reduction of SGA-dosage to minimally sufficient levels [level of evidence: CS, CR]
(III)	Combination of proobsessive SGAs with neutral or antiobsessive SGAs (amisulpride, aripiprazole) in order to reduce the clozapine dosage to minimally sufficient levels [level of evidence: RCT, CS, CR]

Psychotherapy	

	Cognitive behavioural therapy including exposure and response prevention [level of evidence: CS, CR]

Summary of therapeutic approaches for schizophrenia patients with comorbid OCS or OCD. The current level of empirical evidence is indicated in square brackets. CR: case report, CS: case series, and RCT: randomized controlled trial.

## Abbreviations

AMS: Amisulpride
APZ: Aripiprazole
ARMS: At-risk mental state
BDNF: Brain derived neurotrophic factor
CBT: Cognitive behavioural therapy
CLZ: Clozapine
CR: Case report
CS: Case series
DLGAP3: Disks large associated protein 3
fMRI: Functional magnetic resonance imaging
GxEI: Gene and environment interaction
OCS: Obsessive-compulsive symptoms
OCD: Obsessive-compulsive disorder
OFC: Orbitofrontal cortex
OLZ: Olanzapine
SGA: Second generation antipsychotics
SLC1A1: Solute carrier family gene 1A1
SNP: Single nucleotide polymorphism
SSRI: Selective serotonin reuptake inhibitor.
